# Targeting strategies of antenatal balanced energy and protein supplementation in Addis Ababa, Ethiopia: study protocol for a randomized effectiveness study

**DOI:** 10.1186/s13063-024-08002-2

**Published:** 2024-04-30

**Authors:** Dongqing Wang, Tigest Shifraw, Janaina Calu Costa, Semira Abdelmenan, Sitota Tsegaye, Yoseph Berhane, Hanna Gulema, Hanna Berhane, Nebiyou Fasil, Firehiwot Workneh, Workagegnhu Tarekegn, Molin Wang, Nicolas A. Menzies, Alemayehu Worku, Yemane Berhane, Wafaie W. Fawzi

**Affiliations:** 1 Department of Global and Community Health, College of Public Health, George Mason University, Fairfax, VA USA; 2 Department of Global Health and Population, Harvard T.H. Chan School of Public Health, Harvard University, 665 Huntington Avenue, Building 1, Room 1108, Boston, MA 02115 USA; 3 Department of Reproductive Health and Population, Addis Continental Institute of Public Health, Addis Ababa, Ethiopia; 4 Department of Epidemiology and Biostatistics, Addis Continental Institute of Public Health, Addis Ababa, Ethiopia; 5 Department of Nutrition and Behavioral Sciences, Addis Continental Institute of Public Health, Addis Ababa, Ethiopia; 6 Department of Global Health and Health Policy, Addis Continental Institute of Public Health, Addis Ababa, Ethiopia; 7 Department of Epidemiology, Harvard T.H. Chan School of Public Health, Harvard University, Boston, MA USA; 8 Department of Biostatistics, Harvard T.H. Chan School of Public Health, Harvard University, Boston, MA USA; 9 Department of Nutrition, Harvard T.H. Chan School of Public Health, Harvard University, Boston, MA USA

**Keywords:** Study protocol, Effectiveness study, Balanced energy and protein supplementation, Food supplementation, Antenatal nutrition, Gestational weight gain, Ethiopia

## Abstract

**Background:**

Antenatal balanced energy and protein (BEP) supplements have well-documented benefits for pregnancy outcomes. However, considerable practical gaps remain in the effective and cost-effective delivery of antenatal BEP supplements at scale in low- and middle-income countries.

**Methods:**

A randomized effectiveness study will be conducted in two sub-cities of Addis Ababa, Ethiopia, to evaluate the effectiveness, cost-effectiveness, and implementation of different targeting strategies of antenatal BEP supplements. Pregnant women aged 18 to 49, with a gestational age of 24 weeks or less, and attending antenatal visits in one of the nine study health facilities are eligible for enrollment. In six of the health facilities, participants will be randomized to one of three study arms: control (Arm 1), targeted BEP provision based on baseline nutritional status (Arm 2), and targeted BEP supplementation based on baseline nutritional status and monthly gestational weight gain (GWG) monitoring (Arm 3). In the remaining three facilities, participants will be assigned to universal BEP provision (Arm 4). Participants in Arms 2 and 3 will receive BEP supplements if they have undernutrition at enrollment, as defined by a baseline body mass index less than 18.5 kg/m^2^ or mid-upper arm circumference less than 23 cm. In Arm 3, in addition to targeting based on baseline undernutrition, regular weight measurements will be used to identify insufficient GWG and inform the initiation of additional BEP supplements. Participants in Arm 4 will receive BEP supplements until the end of pregnancy, regardless of baseline nutritional status or GWG. All participants will receive standard antenatal care, including iron and folic acid supplementation. A total of 5400 pregnant women will be enrolled, with 1350 participants in each arm. Participants will be followed up monthly during their visits to the antenatal facilities until delivery. Maternal and infant health status will be evaluated within 72 h after delivery and at 6 weeks postpartum. The effectiveness and cost-effectiveness of the different BEP targeting strategies in preventing adverse pregnancy outcomes will be compared across arms. Qualitative data will be analyzed to assess the feasibility, acceptability, and implementation of different supplementation strategies.

**Discussion:**

This study will inform global recommendations and operational guidelines for the effective and cost-effective delivery of antenatal BEP supplements. The targeted approaches have the potential for broader scale-up in Ethiopia and other low-resource settings with a high burden of undernutrition among pregnant women.

**Trial registration:**

ClinicalTrials.gov registration number: NCT06125860. Registered November 9, 2023.

**Supplementary Information:**

The online version contains supplementary material available at 10.1186/s13063-024-08002-2.

## Background

Undernutrition of pregnant women leads to poor reproductive and health consequences for pregnant women; exacerbates the risks of fetal losses, fetal growth restriction, preterm delivery, low birthweight (LBW), and infant morbidity and mortality; and can cause long-term, irreversible, and detrimental cognitive, motor, and health impairments to the offspring [1–3]. Progress in reducing maternal undernutrition has been slow, and improvements have been particularly modest in sub-Saharan Africa and South Asia [3].

In Ethiopia, based on estimates from the 2016 Demographic and Health Surveys (DHS) [4], the prevalence of underweight (body mass index [BMI] < 18.5 kg/m^2^) among women of reproductive age (15 to 49 years) was 22.4%, and the prevalence of anemia was 23.6%, with approximately half of individuals with anemia being moderately or severely anemic. Besides iron-deficiency anemia, women with undernutrition globally are also likely to experience deficiencies in multiple types of other micronutrients simultaneously [5, 6]. Maternal undernutrition in Ethiopia contributes to high burdens of LBW and neonatal and infant mortality. According to the Ethiopia DHS report, in the 5 years preceding 2016, 13.2% of infants were LBW; in the 5 years preceding 2019, 33 per 1000 live births died in the first month and 47 per 1000 live births died in the first year [7].

To address the significant burden of maternal undernutrition, it is critical to ensure appropriate energy and macronutrient consumption and adequate intakes of essential micronutrients to meet the needs of pregnant women and fetuses [8]. Balanced energy and protein (BEP) dietary supplements are foods with less than 25% of the energy from protein [9]. BEP can be provided to pregnant women to supplement their home-based diets. BEP supplements also incorporate essential minerals and vitamins to meet the increasing need for micronutrients during pregnancy.

Accumulating evidence from low- and middle-income countries (LMICs) shows that antenatal BEP supplements reduce the risk of stillbirths and small-for-gestational-age (SGA) births and increase birthweight [9, 10]. A meta-analysis examined the impacts of antenatal BEP supplements in LMICs and reported that the supplementation reduces the risks of stillbirths by 61% (risk ratio [RR] 0.39; 95% confidence interval [CI] 0.19, 0.80), LBW by 40% (RR 0.60; 95% CI 0.41 to 0.86), SGA by 29% (RR 0.71; 95% CI 0.54 to 0.94), and increases mean birthweight by 107.3 g (95% CI 68.5 to 146.0) [10]. The effect on birthweight also appears stronger among pregnant women with undernutrition than those who are well-nourished [9]. BEP supplementation is a particularly promising intervention in sub-Saharan Africa, a region where gender bias and inequities in intra-household food distribution exacerbate women’s inadequate dietary intake [3] and the only region where dietary micronutrient density has declined over the past five decades [11].

Despite the benefits of antenatal BEP supplements, considerable practical gaps remain in the effective and cost-effective delivery of these at scale in resource-poor settings. The antenatal guidelines by the World Health Organization (WHO) recommend that BEP supplements be provided in populations with a high prevalence of undernutrition, usually defined as having a prevalence of underweight of 20% or greater [12]. This universal provision may provide BEP supplements to low-risk pregnant women and may also miss vulnerable pregnant women who may benefit substantially from the intervention but do not reside in a high-risk area. Further, the universal approach can be challenging to implement as few countries meet the criterion at the national level [3], and limited data exist on subnational estimates of underweight.

It has been recognized that BEP targeting strategies based on individual nutritional status may be more impactful and cost-effective than the population-based approach [13]. One suggested strategy of individual targeting is to provide BEP supplements to underweight pregnant women with low BMI or low mid-upper arm circumference (MUAC). A limitation of targeting based on baseline nutritional status is that it does not account for the fact that adequate gestational weight gain (GWG) is also essential for a healthy pregnancy. Inadequate GWG can occur regardless of baseline nutritional status, even among those well-nourished before pregnancy [14]. Inadequate GWG increases the risks of LBW [14–17], prematurity [14, 16, 18], SGA births [14, 15, 17, 18], and neonatal and infant death [19, 20]. A meta-analysis showed that antenatal BEP supplementation increased the weekly rate of gestational weight gain (GWG) by 21 g/week (95% CI 1.5 to 40.0) [21]. Due to its important implications and modifiable nature, GWG is increasingly recognized as an essential target for antenatal monitoring [14]. Therefore, targeting antenatal BEP supplements toward those with inadequate GWG will capture additional pregnant women vulnerable to undernutrition.

Targeted delivery of BEP to pregnant women most likely to benefit from the supplement may have greater cost-effectiveness and be more feasible to implement in resource-constrained settings. There is limited understanding of the effectiveness, cost-effectiveness, and implementation of different strategies of BEP delivery. Filling this gap will inform recommendations and scaling-up of antenatal BEP supplements in LMICs. We will conduct a randomized effectiveness study in Ethiopia, with the following aims: (1) determine the effectiveness of two individual-based antenatal BEP targeting strategies for preventing adverse pregnancy outcomes; (2) compare the cost-effectiveness of the universal BEP provision with two individual-based targeting strategies for preventing adverse pregnancy outcomes; and (3)1 generate implementational evidence on the feasibility and acceptability of different targeting strategies.

## Methods

### Study setting

We will conduct this randomized effectiveness study in Akaki and Nifas Silk-Lafto, two sub-cities of Addis Ababa, Ethiopia. These sub-cities have significant burdens of undernutrition among women of reproductive age. Between 2018 and 2020, among women of reproductive age with a child aged 6 to 59 months, 14.0% in Akaki and 10% in Nifas Silk-Lafto had a BMI less than 18.5 kg/m^2^; 8.6% in Akaki and 11.7% in Nifas Silk-Lafto had a MUAC less than 23 cm; and 17.9% in Akaki and 13.8% in Nifas Silk-Lafto had either a low BMI or a low MUAC (unpublished data).

### Randomization design

**Fig. 1:**
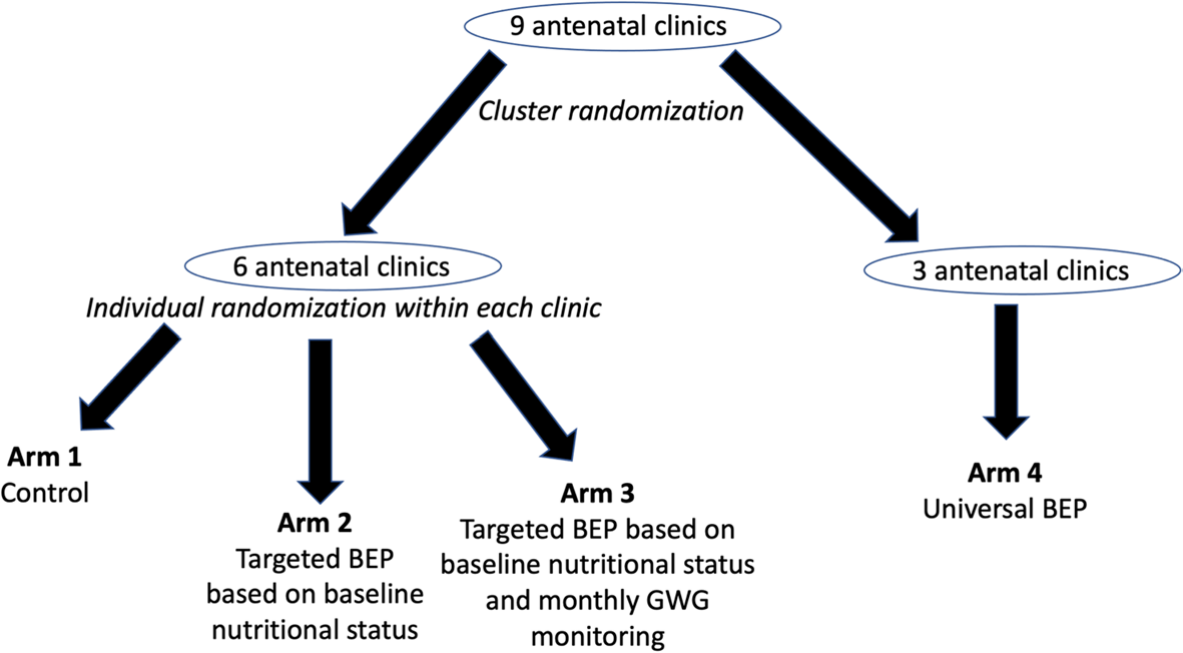
Cluster- and individual-level randomization of a randomized effectiveness study to compare effectiveness, cost-effectiveness, and implementation across different targeting strategies of antenatal balanced energy and protein (BEP) supplementation in Addis Ababa, Ethiopia

We will conduct the study in nine public antenatal health facilities, five in Akaki and four in Nifas Silk-Lafto. This study will include randomization at both cluster level and individual level (Fig. 1). We first conducted cluster randomization among the nine health facilities to allocate three of them to universal BEP (Arm 4). Then, within the remaining six facilities, we will conduct individual randomization within each facility to allocate individual pregnant women to one of three arms: control (Arm 1), targeted BEP based on baseline nutritional status (Arm 2), or targeted BEP based on baseline nutritional status and monthly GWG monitoring (Arm 3). Therefore, all participants in the three antenatal facilities assigned to Arm 4 will receive the same universal BEP intervention. Participants within the other six facilities will receive different interventions based on individual randomization.

To ensure comparability of the three health facilities for Arm 4 versus the six facilities for Arms 1 to 3, we matched the nine antenatal facilities into three triplets before the cluster randomization. Each triplet includes three facilities similar in the annual number of deliveries and the proportion of low birthweight newborns out of all live births. We used coarsened exact matching and the *cem* command [22] in *STATA* 16 (StataCorp, College Station, Texas) for the initial matching, followed by manual fine-tuning to arrive at two matches, with three facilities per match. Then, we randomly assigned one facility out of each triplet to Arm 4. A statistician not directly involved in study implementation conducted the cluster and individual randomization. For the individual randomization, a separate randomization list was generated in permutated blocks for each facility. The study nurses responsible for recruiting and assigning interventions are not aware of the blocking information. Due to the adaptive nature of interventions in this study, blinding the participants and study teams to the assigned interventions will not be possible.

### Screening and enrollment

The inclusion criteria include (1) pregnant women aged 18 to 49; (2) attending antenatal visits in one of the study health facilities; (3) with a gestational age of 24 weeks or less; (4) no known allergies to peanuts or soy; (5) having resided in the current location for at least 12 months; (6) intending to continue antenatal follow-up in the health facility; (7) intending to give birth and remain in the study area until 6 weeks after delivery; and (8) willing to take the BEP supplements for the entire duration of the pregnancy if eligible. We will exclude those above 24 weeks of gestation due to the limited time the participants may receive the intervention and the difficulty of estimating GWG. Criteria 5 to 8 are enacted to restrict to pregnant women most likely to adhere to the allocated intervention and minimize loss to follow-up.

Upon enrollment, trained study nurses will collect information on demographic characteristics, socioeconomic status, household food insecurity, health conditions, and reproductive history using interviewer-administered questionnaires. Study nurses will measure height, weight, MUAC, and blood pressure; assess fingerstick hemoglobin concentration; and conduct a dipstick proteinuria test.

### Gestational age assessment and quality assurance

At the enrollment visit, women will provide a self-report of their last menstrual period (LMP). Those with an LMP between 8 and 24 weeks are eligible for the study and will receive an ultrasound assessment for pregnancy confirmation and gestational age dating. If the ultrasound shows less than 8 weeks gestation, the participants will be offered another ultrasound in 4 weeks. For women who cannot recall their LMP despite support from the study nurses, they will be provided with the ultrasound assessment at enrollment, and the ultrasound-based gestational age estimate will be used for eligibility confirmation. During data analysis, we will triangulate the gestational age estimates based on self-report and ultrasound assessment to determine the best obstetric estimate for the relevant outcomes (e.g., preterm birth and SGA). Two study nurses were trained to perform the ultrasound scans at each health facility.

An external sonographer will review a random sample of the ultrasound scan images weekly. Each image will be scored based on a pre-specified scoring spreadsheet with five domains: crown-rump length, biparietal diameter, head circumference, abdominal circumference, and femur length. Each domain has a score ranging from 0 to 7, with the total possible score ranging between 0 and 35. The external sonographer will also provide written comments for each image to offer specific and individualized feedback. In addition to the external quality assurance, we will provide quarterly reinforcement training and standardization for all study nurses responsible for ultrasound scans.

### Pregnancy follow-up

We will assess each participant every 4 weeks during their visits to the antenatal facilities until delivery, death, or fetal loss, whichever occurs first. Data collected during the monthly follow-up visits will include pregnancy status, anthropometry, and clinical assessments. The clinical assessments will include a physical examination, blood pressure measurement, collection of morbidity symptoms, and dipstick proteinuria test. During 28–32 weeks of gestation, we will conduct another fingerstick assessment of hemoglobin concentration. We will conduct a food frequency questionnaire during 28–32 weeks of gestation. We will conduct 24-h dietary recalls once in the second trimester during 14–28 weeks of gestation and once in the third trimester after 32 weeks of gestation.

### Delivery assessment

We will assess delivery outcomes within 3 days (< 72 h) of birth, regardless of whether a participant delivers in a health facility or at home. An outcome assessment team will contact participants weekly after 36 weeks of gestation to maintain good rapport with the participant and ensure that the delivery information will be recorded promptly. We will also give participants the phone number of a study-specific ‘birth hotline’ so they can report their deliveries. When the outcome assessment team is alerted of a potential birth in advance, they will record delivery details, including vital status and anthropometry. For deliveries at home, the outcome assessment team will visit the home at the earliest possible time and no later than 72 h after birth to assess the vital status and measure the newborn’s anthropometry, including weight, length, and head and chest circumference.

### Postnatal follow-up

The outcome assessment team will visit all study participants at home or in the health facility at 6 weeks postpartum to assess maternal and infant vital and health status and perform maternal and infant anthropometric measurements.

### Interventions

**Fig. 2:**
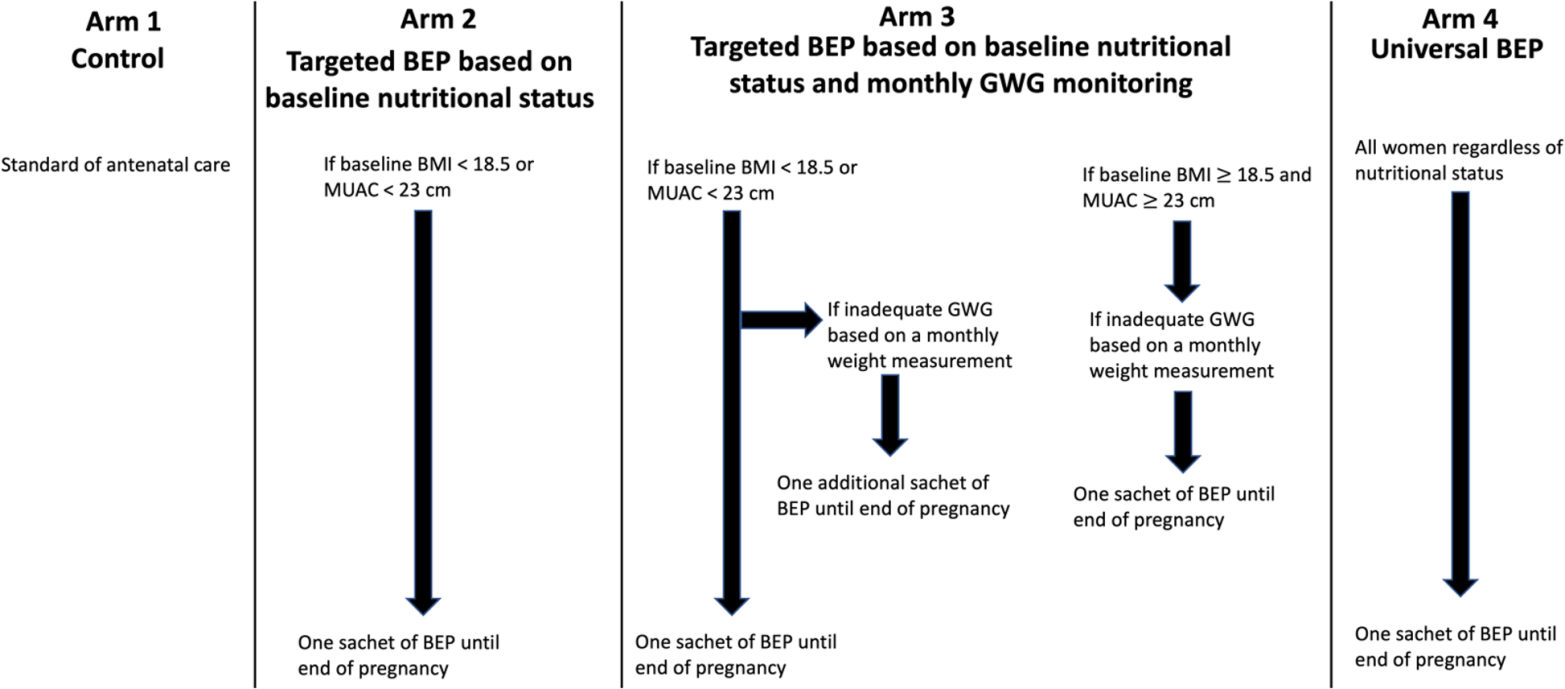
Study design of comparing different targeting approaches of antenatal balanced energy and protein supplementation in Addis Ababa, Ethiopia. BEP, balanced energy and protein; BMI, body mass index; GWG, gestational weight gain; MUAC, mid-upper arm circumference

The participants will be randomized to four study arms (Fig. 2). Participants in all four arms will receive the standard of antenatal care per the WHO antenatal guidelines [12] and the Ethiopian antenatal guidelines, including iron and folic acid supplementation. Arm 1 will be the control arm and will only receive this standard of care.

Participants in Arm 2 (targeted BEP based on baseline nutritional status), in addition to the standard of care, will receive BEP supplements if their baseline BMI is less than 18.5 kg/m^2^ or their baseline MUAC is less than 23 cm. The cutoff of 18.5 kg/m^2^ for BMI corresponds to the WHO definition of underweight [23]. The cutoff of 23 cm for MUAC is selected based on prior literature [24, 25] and analyses of the predictive power of adverse pregnancy outcomes based on internal unpublished data, prioritizing specificity over sensitivity to reduce false positives (i.e., so fewer participants would receive the supplements unnecessarily). MUAC is relatively easy to measure; hence, it has the potential of complementing BMI for screening high-risk pregnant women in need of targeted interventions [26]. Weight gain during the first trimester of pregnancy is minimal, and most of the GWG occurs during the second and third trimesters [14]. Therefore, for participants enrolled before the end of the first trimester (13^6/7^ weeks), we will use the weight measured at enrollment as the proxy for pre-pregnancy weight. For participants enrolled during the second trimester (but no later than 24 weeks of gestation), we will impute early-pregnancy gestational weight, as further described below. We will use the baseline MUAC measurement regardless of gestational age at enrollment. Participants who meet either the low BMI or the low MUAC criterion will receive the BEP supplements until the end of their pregnancy.

Participants in Arm 3 (targeted BEP based on baseline nutritional status and monthly GWG monitoring), in addition to the standard of care, will receive BEP supplements if their baseline BMI is less than 18.5 kg/m^2^ or their baseline MUAC is less than 23 cm, in the same way as described for Arm 2. Additionally, we will use monthly measurements of GWG to inform the initiation of additional BEP supplements for those with inadequate GWG. If the participant is already on antenatal BEP supplements initiated based on baseline undernutrition, she will receive additional BEP supplements on top of the ration she is already receiving. If the participant does not have undernutrition at baseline and thus is not already on the supplements, she will start receiving BEP supplements from that visit in which inadequate GWG was identified. The supplementation initiated based on inadequate GWG will be provided until the end of pregnancy regardless of gestational weight gain assessments during subsequent visits.

All participants in Arm 4 (universal BEP) will receive BEP supplements until the end of pregnancy, regardless of baseline nutritional status or GWG. This arm mimics the universal BEP supplementation as currently recommended by the WHO. For participants with overweight or obesity (i.e., BMI $\ge$ 25 kg/m^2^) at enrollment during the first trimester, or based on imputation for those enrolled in the second trimester, we will stop the provision of supplements if they start to gain excessive GWG. Definitions of inadequate and excessive GWG are provided below.

### Balanced energy and protein supplements

We will use Plumpy’Sup as the BEP supplement. The product is a smooth, homogeneous, thick, dark-brown-colored paste containing peanuts, sugar, oil, milk powder, and vitamin and mineral premix. It is ready to use and can be squeezed out of the sachet and eaten directly without cooking, mixing, or diluting. Each 100-g sachet of Plumpy’Sup includes 540 kcal of energy, 12 g of protein, and an array of micronutrients. The nutrient composition of the supplement aligns with the recommended specifications for the nutritional composition of BEP based on the expert consultation held by the Bill & Melinda Gates Foundation (Table 1) [27].

**Table 1:** Nutrition composition of Plumpy’Sup and expert recommendation from the Bill & Melinda Gates Foundation

	Plumpy’Sup / 100-g sachet	BMGF expert recommendation for BEP [27]
Energy	540 kcal	250–500 kcal
Protein	12 g	14–18 g
% energy from protein	9.0%	No recommendation
Lipids	33 g (55% energy)	10–60%
Calcium	630 mg	500–1000 mg
Phosphorus	610 mg	300–700 mg
Potassium	980 mg	2000–5100 mg
Magnesium	175 mg	145–350 mg
Manganese	1.3 mg	2.1–2.6 mg
Sodium	180 mg	No recommendation
Vitamin K	27 $\mu$ g	72–90 $\mu$ g
Biotin	60 $\mu$ g	28–35 $\mu$ g
Pantothenic acid	4.0 mg	5.6–7.0 mg
Zinc	12 mg	15–20 mg
Copper	1.4 mg	1.0–1.3 mg
Iron	11 mg	22–27 mg
Iodine	140 $\mu$ g	209–290 $\mu$ g
Selenium	20 $\mu$ g	60–70 $\mu$ g
Vitamin A	750 $\mu$ g RE	550–770 $\mu$ g
Vitamin D	15 $\mu$ g	10–15 $\mu$ g
Vitamin E	16 mg	16–19 mg
Vitamin C	60 mg	100–120 mg
Vitamin B1	1.0 mg	1.2–1.4 mg
Vitamin B2	2.1 mg	1.3–1.6 mg
Niacin	13 mg	14–18 mg
Vitamin B6	1.8 mg	1.7–2 mg
Folic acid	330 $\mu$ g	400–600 $\mu$ g
Vitamin B12	2.7 $\mu$ g	2.4–2.8 $\mu$ g

*BEP* balanced energy and protein, *BMGF* Bill & Melinda Gates Foundation, *RE* retinol equivalent

### Distribution of nutritional supplements and assessment of compliance

The BEP supplements will be distributed by study nurses at baseline visits and every month when participants come to the antenatal facility for follow-up visits. In addition to the amount determined by the intervention assignment, we will also provide a modest quantity of extra supplements to compensate for the practice of intra-household sharing. We determined the amount to compensate for intra-household sharing through a formative period. We will assess compliance with the assigned intervention by requesting participants to bring back unused sachets and packages of used sachets during their monthly follow-up visits. We will also directly ask the participants about the use of the supplements in the previous week and since the last visit.

### Early-pregnancy weight imputation and gestational weight gain monitoring

A significant proportion of pregnant women in practical settings do not present themselves at antenatal facilities until later in pregnancy, typically during the second trimester. Therefore, to approach a real-world setting to the greatest extent possible, we will also include participants enrolled during the second trimester of pregnancy. However, gestational weight beyond the first trimester does not reflect the pre-pregnancy or early-pregnancy nutritional status. It thus cannot be used as a proxy for baseline nutritional status or as an anchor point for the calculation of GWG. Therefore, for participants enrolled during the second trimester of pregnancy (but no later than 24 weeks of gestation), we will impute their early-pregnancy gestational weight.

As part of the preparatory activities, we developed and validated an imputation equation that estimates a pregnant woman’s weight during early pregnancy, using individual characteristics and gestational weights collected later during pregnancy. We used individual characteristics, including current gestational weight, current gestational age, and height, as potential predictors. We developed the equation using the GWG Pooling Project data, which combined data from 55 studies and approximately 150,000 pregnant women in LMICs [28, 29]. The GWG Pooling Project has accumulated a wealth of data on longitudinal gestational weights, including pre-pregnancy weights from some studies and early-pregnancy weights from many studies [28, 29]. We conducted external validation of the tool using internal data from Ethiopia [30].

We incorporated the imputation algorithm into the electronic tool for data collection. The imputation equation estimates a pregnant woman’s early-pregnancy weight, an essential quantity for evaluating baseline nutritional status and a critical anchor point for assessing GWG adequacy. The electronic tool also has the built-in capacity of monthly GWG adequacy monitoring based on the Institute of Medicine (IOM) recommendations and the (measured or imputed) early-pregnancy weight.

### Quantifying gestational weight gain adequacy

Inadequate weight gain at each visit will be quantified using the GWG percent adequacy ratio, calculated as the ratio of the observed GWG to the weight gain recommended by the IOM based on pre-pregnancy BMI [14]. First, we will calculate the observed GWG as the difference between each weight measurement and the early-pregnancy weight (measured for those enrolled during the first trimester or imputed for those enrolled during the second trimester). Second, we will estimate the IOM-recommended GWG at the time of the weight measurement. Finally, we will calculate the percent adequacy by dividing the observed by the recommended GWG. The formulas are shown below.

$$\mathrm{GWG\,percent\,adequacy }(\mathrm{\%}) =\frac{\mathrm{Observed\,GWG}}{\mathrm{Recommended\,GWG}}\times 100\%$$

$$\mathrm{Observed\,GWG}=\text{weight at follow-up visit}-\mathrm{first \,measured \,or \,imputed \,weight \,measure}$$

$${\text {Recommended GWG}}=\left(\frac{{\text{Expected Trimester}}\;1\;{\text {GWG}}}{13.86\;{\text{weeks}}}\right)\left(13.86\;{\text{weeks}}-{\text{gestational age at first measured or imputed weight measure}}\right)+\left({\text{gestational age at follow-up visit}}-13.86{\text{wks}}\right)\times {\text{recommended weekly rate of GWG for trimester}}\;2\;\& \ {\text{trimester}}\;3$$

The expected first-trimester GWG will be 2, 1, and 0.5 kg for pregnant women with underweight/normal weight, overweight, and obesity, respectively. The recommended weekly rate of GWG in the second and third trimesters will be 0.51 kg/week for underweight, 0.42 kg/week for normal weight, 0.28 kg/week for overweight, and 0.22 kg/week for obesity [14].

GWG adequacy ratio accounts for the gestational age at each weight measurement and takes advantage of the well-established IOM recommendations. To account for the allowable range specified in the IOM standards and in accordance with previous studies [28, 29, 31–33], we will define inadequate GWG at each visit as a percent adequacy ratio less than 90%, adequate GWG as a percent adequacy ratio between 90 and 125%, and excessive GWG as a percent adequacy ratio greater than 125%.

### Formative research

During the preparatory phase of the study, we conducted formative research for a preliminary evaluation of the potential acceptability of the BEP intervention. We established an initial understanding of potential facilitators and barriers to the continued use of BEP supplements. Forty-five participants were selected purposively from the nine health centers and provided with BEP sufficient for 4 weeks with clear instructions. After this period, we conducted in-depth interviews (IDIs) with 39 participants. We used a structured framework during the IDIs to elicit contextual data relevant to pregnant women’s general perspectives on dietary preferences and culinary practice. The framework included questions about factors that may influence the acceptability and continued use of the BEP supplements, such as flavor preferences, food-sharing dynamics, and local food environments.

### Cost data collection

We will focus on three major cost categories for empirical cost data collection: (a) direct costs required to provide each intervention; (b) costs or cost-savings resulting from changes in utilization for health services that would plausibly be impacted by the intervention, such as medical care for participants with pregnancy complications; and (c) costs and cost-saving borne by patients, as could results from seeking and receiving related medical care, as well as productivity losses due to pregnancy-related morbidity and accessing healthcare.

For intervention costs, we will use an ingredients approach [34] to identify and cost all resources required for the targeted and universal approaches of BEP delivery. The costs will be broadly composed of four categories, including personnel time (e.g., nurses, sonographers); BEP supplements (cost of products and shipping); medical equipment and supplies (e.g., ultrasound machines and anthropometric measurement tools); and infrastructure and logistical support (e.g., storage space for BEP supplements). For each category, we will create data collection instruments to record resource utilization and use these to quantify the resources required throughout pregnancy. We will review financial records and conduct key informant interviews at each facility to estimate resource requirements if the program is to be scaled up.

For costs due to changes in utilization of related health services, we will conduct interviews to identify health services plausibly affected by the interventions (i.e., by changing the probability of different pregnancy outcomes). Based on these interviews, we will identify the relevant services and collate information on the utilization of these services for each study arm. We will collect cost data to calculate unit costs for each service and multiply them by recorded service utilization to estimate total costs per patient for related health services, for each study arm.

For patient-incurred costs, we will conduct interviews with a sub-sample of study participants to understand the direct medical and non-medical costs incurred to participate in the interventions and receive related services. In the same survey, we will collect information on time spent participating in interventions (beyond the routine antenatal process), as well as time spent unable to work or contribute to household activities due to pregnancy-related morbidity, to estimate productivity losses.

### Qualitative evaluation

Toward the end of the study, we will use focus group discussions (FGDs) and IDIs to generate qualitative implementational evidence regarding the feasibility and acceptability of different modalities of antenatal BEP supplementation and the facilitating and inhibitory factors of uptake and continued use of the supplements.

The qualitative evaluation will be conducted from the perspectives of pregnant women and healthcare providers. We will conduct FGDs among pregnant women participating in the targeted or universal BEP intervention. We will select eight participants from each of the nine antenatal health centers to form the focus group for that facility. Therefore, the total number of participants for the FGDs will be 72 pregnant women (eight participants per facility from nine facilities). For the six facilities with a mixture of participants of Arms 2 and 3, we will ensure that participants in both targeted arms will be represented in the focus group and will only include participants who received the supplements under the corresponding targeting criterion. We will also invite one study nurse from each antenatal facility to form a separate focus group with nine nurses. The study nurses for the FGDs will be selected from those closely involved with the day-to-day study activities, including the distribution of the BEP supplements.

We will develop semi-structured FGD questions and guides for pregnant women and healthcare providers. The FGDs with pregnant women will cover a range of thematic topics, including general questions on household food practices and beliefs, diet during pregnancy and lactation, availability and access to nutritious foods, antenatal care-seeking behaviors, and supplement-specific questions, including experience with product taste, product preparation, levels and barriers of adherence and continued use, dynamics of intra-household sharing, and interface of the BEP products with local food environments. The FGDs with nurses will cover the burdens, enablers, and barriers of targeted or universal distribution for healthcare providers and their perspectives on sustainability, buy-in, and scale-up. All FGD questions and guides will be pretested and modified prior to implementation.

The FGDs will be held at the antenatal facilities and last approximately 2 to 2.5 h. We will use private rooms perceived as neutral as possible. Participants will be encouraged to speak in the language in which they are most comfortable and confident. The FGDs will be audio-recorded and transcribed from the local language into English. The focus groups will be audio-recorded and conducted with one moderator (a trained research assistant) and one notetaker who will support with additional observational notes that may not be captured through the audio recording.

In addition to FGDs with pregnant women and nurses, we will conduct one-on-one, IDIs with the head administrator of each of the nine antenatal facility. The interviews with the administrators will elicit additional information on the healthcare providers’ experience with various modalities of supplement delivery and understand their perspectives on sustainability, buy-in, and scale-up of targeted or universal supplementation. The IDIs with the head administrators will last approximately 60 min and be conducted by a trained research assistant supported by an additional notetaker in their antenatal facilities.

The expected participant timeline is illustrated in Table 2. The study protocol was designed and developed in accordance with the Standard Protocol Items: Recommendations for Interventional Trials (SPIRIT) 2013 checklist (Additional file 1, SPIRIT Checklist).

**Table 2:**
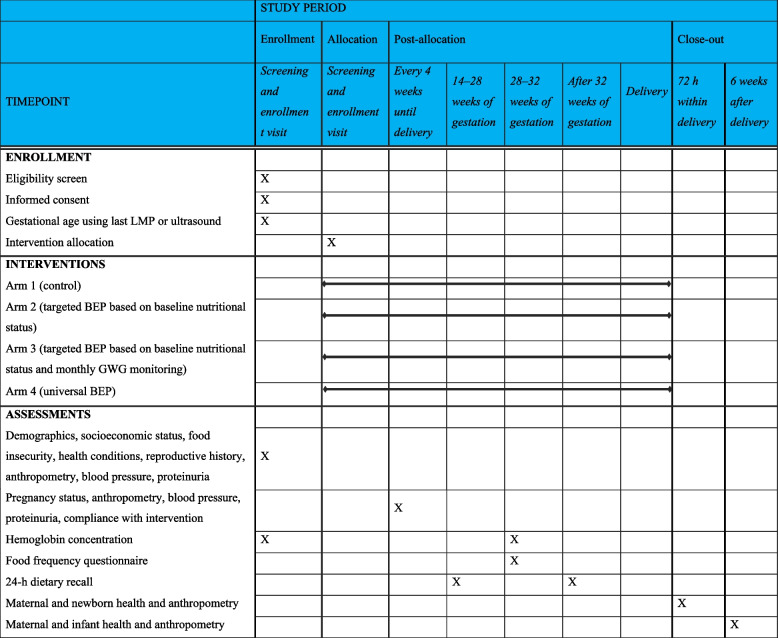
Schedule of enrollment, intervention, and assessments based on SPIRIT guidelines^1^

*BEP* balanced energy and protein, *GWG* gestational weight gain, *LMP* last menstrual period

### Sample size calculation

We used SGA as the primary outcome to calculate the required sample size, as evidence suggests that one of the primary effects of antenatal BEP supplements is to reduce the risk of SGA [9]. We used a two-sample test of proportion to conduct sample size calculation, comparing Arm 2 to Arm 1, and comparing Arm 3 to Arm 1, individually. We used the Power and Sample Size Calculation Program by Dupont and Plummer to conduct the calculation [35]. The following assumptions were used:

1. SGA incidence of 30% in the control group based on unpublished data from the Akaki or Nifas Silk-Lafto sub-cities;
2. An effect size (risk ratio) of 0.80 based on the Cochrane systematic review by Ota et al. (2015) [9];
3. A 14% rate of loss to follow-up;
4. A two-sided significance level of 0.05 and a power of 90%.

Under these assumptions, the target sample size per arm is 1337 pregnant women, and the total number of participants for Arms 1 to 4 would be 5348 participants (i.e., 1337 × 4), or 5400 after rounding to the next hundred. Therefore, we will enroll 5400 pregnant women in this study. Additional file 2 shows the detectable RRs given alternative assumptions of SGA incidence and statistical power.

### Data management and monitoring

The Addis Continental Institute of Public Health (ACIPH) will be responsible for data collection and management. Data will be electronically collected using pre-programmed questionnaires and tools on research tablets. All data collected as part of the study will be uploaded and stored in a secure server at ACIPH, with access restricted to authorized members of the study team only. Data will be stored in coded format, stripped of any identifiers and with the linkage key stored separately. Any paper forms will be stored in locked cabinets that are accessible only to authorized members of the study team. De-identified data will be transferred to Harvard T.H. Chan School of Public Health for joint monitoring, including the examination of data values for outliers and potential errors.

### Data analysis

#### Effectiveness analysis

We will evaluate the effectiveness of the individual-based targeting strategies by comparing the incidences of adverse pregnancy outcomes across arms, using Arm 1 (control group) as the reference. We will use SGA as the primary outcome. The secondary outcomes will include inadequate and excessive GWG close to delivery, stillbirths, preterm births, LBW, macrosomia, large-for-gestational-age births, anemia during the third trimester, pregnancy-related death, neonatal death, and perinatal death, as defined in Table 3. We will use log-binomial models to calculate RRs and the 95% CIs for the following comparisons: (1) Arm 2 versus Arm 1; (2) Arm 3 versus Arm 1; (3) Arm 4 versus Arm 1; and (4) Arm 2 versus Arm 3. We will use modified Poisson models with robust variance estimates to achieve model convergence whenever necessary [36]. We will conduct the primary analyses using the intention-to-treat approach. The outcomes will be compared across arms among all participants in each arm, regardless of whether they meet the criteria to receive BEP, so we can quantify the impacts of the targeting strategies at the population level, not only merely among those who received the supplementation.

**Table 3:** Definition of primary and secondary outcomes

Outcomes	Definition
**Primary outcomes**	
Small-for-gestational-age births	Live birth whose birthweight for sex and gestational age is < 10th percentile based on the INTERGROWTH-21st standards
**Secondary outcomes**	
Inadequate gestational weight gain close to delivery	Gestational weight gain percent adequacy ratio less than 90% at the last weight measurement before delivery
Excessive gestational weight gain close to delivery	Gestational weight gain percent adequacy ratio greater than 125% at the last weight measurement before delivery
Stillbirth	Fetal death between 28 weeks of gestation and delivery
Preterm birth	Live birth < 37 completed weeks of gestation
Low birthweight	Live birth weighing < 2500 g
Macrosomia	Live birth weighing > 4000 g
Large-for-gestational-age births	Live birth whose birthweight for sex and gestational age is > 90th percentile based on the INTERGROWTH-21st standards
Third-trimester anemia	Hemoglobin concentration < 11 g/dL during $\ge$ 28 weeks of gestation
Neonatal death	Death of live newborn < 28 days of life
Perinatal death	Fetal death between 28 weeks of gestational age and delivery, or newborn death < 7 days of life

We will investigate the modification of the effect of the targeted intervention on primary and secondary outcomes by potential effect modifiers at baseline, including maternal age, dietary intake, anemia status, and reproductive health history. We will explore effect modification by including interaction terms in regression models and assess statistical significance using likelihood ratio tests. We will conduct the analyses for effectiveness using SAS 9.4 (SAS Institute Inc., Cary, North Carolina) at a two-sided $\alpha$ level of 0.05.

#### Cost-effectiveness analysis

We will use the cost data and effectiveness results to conduct an incremental cost-effectiveness analysis comparing the four intervention approaches [37, 38]. We will develop a simple decision-analytic model to extrapolate long-term health outcomes, including changes in survival, quality-of-life, and disability-adjusted life years (DALYs), based on the empirical endpoints of the study. We will use this model to estimate the health losses associated with each study outcome. We will apply these values to the distribution of outcomes obtained for each study arm. We will summarize these differences as life years gained and DALYs averted by the study arms relative to each other.

We will assess cost-effectiveness from a range of perspectives, including provider, the health system, and societal. The first primary cost-effectiveness endpoint will be the incremental cost per instance of SGA averted, matching the primary endpoint of the effectiveness evaluation. The second primary cost-effectiveness endpoint will be the incremental cost per DALY averted, based on the results of the decision-analytic modeling. While this outcome will require additional assumptions beyond the data collected in the study, it is used for many cost-effectiveness analyses of health interventions, allowing us to compare cost-effectiveness results to other health interventions and established cost-effectiveness benchmarks [39]. Secondary cost-effectiveness endpoints will be the incremental cost per adverse pregnancy outcome averted (the union of adverse outcomes in Table 3) and the incremental cost per life year saved (based on the results of the decision-analytic modeling). We will use established methods to describe uncertainty in cost-effectiveness results [40].

#### Qualitative analysis

Qualitative data collected from the FGDs and IDIs will be analyzed using thematic analysis. The thematic analytical approach allows for the systematic identification and analysis of patterns and themes within a qualitative dataset [41]. This inductive process begins as soon as data (i.e., interview transcripts and field notes from the FGDs and IDIs) become available. We will begin with an “open coding” process where the qualitative data are explored without making prior assumptions about what might be discovered. Dominant, recurring themes will be identified through the review of transcripts and field notes, and we will develop a thematic framework iteratively [42]. Salient concepts will then be coded, and all transcripts will be imported for analysis by labeling phrases within the transcripts. A random selection of the transcripts will be independently reviewed by two researchers in the study team to cross-check codes, ensure consistency, and maintain quality assurance.

The emerging trends from the qualitative coding will be critically analyzed to ensure that the emerging themes are relevant to the research objectives [43], namely: household food practices and beliefs, diet during pregnancy and lactation, availability and access to nutritious foods, antenatal care-seeking behaviors, and supplement-related themes including experience with product taste, product preparation, levels and barriers of adherence and continued use, dynamics of intra-household sharing, and interface of the BEP products with local food environments, and the healthcare providers’ perspectives on sustainability, buy-in, and scale-up. We will conduct the coding and analyses of the qualitative data using the Dedoose analytic software (Version 8.2.32; SocioCultural Research Consultants, 2016).

### Training, standardization, and monitoring

Before study implementation, all study staff will be trained in the study objectives, intervention design, and good clinical practice. Study staff will also undergo intensive training in study processes such as consenting, anthropometry measurements (including the monthly evaluation of GWG), BEP supplements provision, and pregnancy outcomes assessment. Inter- and intra-observer standardization exercises for all anthropometry measures will be conducted and repeated every 6 months during the study implementation period. Weighing scales, length board, MUAC tape, and digital blood pressure monitor will be routinely calibrated using standard measurement equipment.

The adverse events (AEs) and serious adverse events (SAEs) considered for this study will include miscarriage, stillbirth, maternal deaths, infant deaths, maternal hospitalization (for any reason except hospitalization for childbirth), and infant hospitalization (for any reason). The occurrence of these AEs/SAEs will be ascertained through the routinely collected data. The AEs and SAEs will be reported to IRB as part of the progress reports annually. The study principal investigators are responsible for reporting these AEs/SAEs to the institutional review boards in the United States and Ethiopia at the time of annual review. In the unlikely event of harm resulting from participation in this trial, we will review these incidents to determine if they are related to trial participation. In cases where harm is determined to be directly attributed to trial participation, we will facilitate the necessary medical care for the participants.

We formulated a technical advisory group (TAG) consisting of four senior experts on epidemiology and nutrition. Prior to the start of the study, we consulted with the TAG for their technical input on various study design features including the definitions of inadequate and excessive GWG. A clinical trial expert external to the study will be invited to conduct external trial auditing on a regular basis.

### Ethical considerations

This study was approved by the Institutional Review Board at Harvard T.H. Chan School of Public Health (Protocol #: IRB22-1245) and the Institutional Ethical Review Board of Addis Continental Institute of Public Health (Ref: ACIPH/IRB/008/2022). The study is registered at ClinicalTrials.gov (registration number: NCT06125860). Important protocol modifications (e.g., changes to eligibility criteria, outcomes, analyses) will be communicated to these Institutional Review Boards and the clinical trial registry at ClinicalTrials.gov.

Written individual informed consent in the local language of Amharic will be obtained by study nurses from eligible pregnant women before their inclusion in the study. The form will be read aloud to participants who cannot read. For those who cannot sign, a thumb imprint will be taken and witnessed by an impartial literate witness.

## Discussion

This study will establish the effectiveness and cost-effectiveness and generate implementational evidence of different targeting strategies of antenatal BEP supplements. The study will address critical implementation questions regarding the effective and cost-effective delivery of these supplements to pregnant women in resource-poor settings. This study challenges the current paradigm of non-targeted nutrition interventions and advances precision public health in low-resource settings for maximal impacts with optimized resource allocations. We expect the findings of this study to inform global recommendations and operational guidelines for BEP delivery.

Throughout this study, we will engage with stakeholders at different levels to keep them informed about study progress. We have already organized multiples meetings with local stakeholders including senior officials from the Ministry of Health of Ethiopia and the municipal government of Addis Ababa. We have devised multimodal dissemination of the findings, seeking to share findings in different formats with a broad constituency of stakeholders. We plan to prepare multiple peer-reviewed manuscripts that document findings from the quantitative analysis on effectiveness and cost-effectiveness and findings from the qualitative analysis on implementational evidence. We will also create an evidence package that summarizes key study findings. We will disseminate our findings to the Ethiopian government and discuss potential options for scaling up and integrating the targeted antenatal BEP delivery into Ethiopia’s healthcare systems and social safety net programs. As maternal undernutrition presents a serious challenge not only Ethiopia but also many other sub-Saharan African countries [3], we will also share the evidence package with stakeholders at the international level including the WHO, the United Nations Children’s Fund, and the World Food Programme for the evidence to inform global guidelines.

This study will fill the critical gaps in the effective and cost-effective provision of BEP supplements to pregnant women in LMICs. It is our expectation that the targeting approach with the greatest effectiveness and cost-effectiveness will have the potential for scale-up in Ethiopia and other low-resource settings in sub-Saharan Africa that have a high burden of maternal undernutrition. By targeting pregnant women most in need and those most likely to benefit, this study will ultimately contribute to the improvement of maternal nutrition and the reduction in health disparity.

## Trial status

Protocol version number: 4.0. Protocol version date: October 27, 2023. The recruitment of participants started on August 7, 2023. The approximate date when recruitment will be completed is February 5, 2025.

### Supplementary Information

**Supplementary Material 1. **

**Supplementary Material 2. **

## Data Availability

Individual participant data cannot be shared publicly. A data transfer agreement between Harvard T.H. Chan School of Public Health and Addis Continental Institute of Public Health stipulates that data will be kept confidential and will not be shared beyond the research teams without prior permission. The de-identified dataset supporting this research may be made available following a request submitted to the corresponding author and be granted after obtaining permissions from the Institutional Ethical Review Board of Addis Continental Institute of Public Health.

## Abbreviations

AE: Adverse event
ACIPH: Addis Continental Institute of Public Health
BEP: Balanced energy and protein
BMI: Body mass index
CI: Confidence interval
DALY: Disability-adjusted life year
DHS: Demographic and Health Surveys
FGD: Focus group discussion
GWG: Gestational weight gain
IDI: In-depth interview
IOM: Institute of Medicine
LBW: Low birthweight
LMICs: Low- and middle-income countries
LMP: Last menstrual period
MUAC: Mid-upper arm circumference
RR: Risk ratio
SAE: Serious adverse event
SGA: Small for gestational age
WHO: World Health Organization
