# Supplementary material for: Targeting strategies of antenatal balanced energy and protein supplementation in Addis Ababa, Ethiopia: study protocol for a randomized effectiveness study
Source: Trials. 2024 Apr 30;25:291. doi: 10.1186/s13063-024-08002-2 (PMC11059725; doi:10.1186/s13063-024-08002-2)
Supplement: Supplementary file 2 — Supplementary Material 2. [file 13063_2024_8002_MOESM2_ESM.docx]

**Additional file 2.** Detectable effect sizes under different assumptions of small-for-gestational-age birth incidence and statistical power

| Detectable risk ratio | | Statistical power | | |
| --- | --- | --- | --- | --- |
|  |  | 90% | 85% | 80% |
| Incidence of small-for-gestational-age birth in the control arm | 20% | 0.744 | 0.762 | 0.777 |
|  | 22% | 0.757 | 0.775 | 0.789 |
|  | 24% | 0.770 | 0.786 | 0.800 |
|  | 30% | **0.800** | 0.815 | 0.826 |
|  | 33% | 0.812 | 0.826 | 0.837 |
|  | 37% | 0.827 | 0.840 | 0.850 |
